# Split doses versus whole dose bowel preparation using polyethylene glycol for colonoscopy: A multicentric prospective Lebanese randomized trial between 2021 and 2023

**DOI:** 10.1002/hsr2.2047

**Published:** 2024-04-22

**Authors:** Blaybel Sara, Hammoud Ghinwa, Mourda Layla, Hallal Mahmoud, Khalil Ali, Mckey Remy

**Affiliations:** ^1^ Department of Internal Medicine, Faculty of Medical Sciences Lebanese University Hadat Lebanon; ^2^ Department of Gastroenterology and Hepatology Al Zahraa Hospital University Medical Center (Zhumc) Beirut Lebanon; ^3^ Department of Gastroenterology Lebanese University Beirut Lebanon

**Keywords:** bowel preparation, colonoscopy, polyethylene glycol, split doses, whole dose

## Abstract

**Background and Aims:**

Bowel preparation is considered as major obstacle before colonoscopy, and it is often reported as the most feared part of the procedure. The aim of this study is to determine the difference in efficacy between a split dose of PEG and the previous day regimen in cleaning the colon, using Boston bowel preparation scale. In addition, also to evaluate patient satisfaction regarding the modality of preparation.

**Methods:**

The study included 200 hospitalized patients undergoing colonoscopy at Beirut hospitals between 2021 and 2023. One of the two regimens will be prescribed randomly to the patients before colonoscopy: 98 (49%) in Group A (patients treated with PEG preparation as a split dose for 2 days), and 102 (51%) in Group B (patients taking PEG preparation as a whole dose). Data was analyzed using SPSS version 25.

**Results:**

Patients were distributed between 105 (52.5%) males and 95 (47.5%) females. The top two indications for colonoscopy were bleeding (34%), change in bowel habits (constipation/diarrhea) (32%). Patients experienced adverse events noting cramps (48.5%), stomach ache (32%), headache (31%), vomiting (53%), nausea (53%), sleep disturbance (27%), bloating (26.5%), and malaise (26%). A statistically significant difference (*p* = 0.040) was detected in sleep disturbance: 20.4% of patients in group A and 33.3% of patients in group B. The average satisfaction score was 3.02 ± 1.03 over 4 (Group A) and 3.04 ± 0.99 over 4 (Group B) (*p* = 0.896). The average BBPS was 8.07 ± 1.14 (Group A) and 8.28 ± 1.0 (Group B) (*p* = 0.162).

**Conclusion:**

The two administrations were almost similar in term of satisfaction and BBPS. As multiple factors like age, sexe, comorbidities may contribute in altering how much a given drug is safe and efficace, more research is needed to choose the best 3regimen for each patient.

## 1 PART I: INTRODUCTION

### 1.1 General overview

Colonoscopy is a standard way to diagnose and treat some colonic pathologies. The need for colonoscopy is constantly rising as colorectal cancer (CRC) screening and monitoring expand globally.^1^ To obtain an accurate diagnosis and effective treatment, a detailed visualization of the colonic mucosa is necessary; therefore, bowel preparation before colonoscopy is an integral part of the process.^2^ However, bowel preparation is considered as major obstacle for patients requiring a colonoscopy, and it is often reported as the most feared part of the procedure.^3^

Unfortunately, unsatisfactory bowel cleansing is found in certain proportion (about 10%–25%) of colonoscopies which can result in higher procedure hours, missed lesions, lower cecal intubation rates, and repeated procedure at more frequent intervals.^2^, ^3^ Therefore, accurate and reliable colonoscopic findings are highly dependent on a good preparation.^4^ This preparation should not only comprise cleaning of the colon, but also patient tailored volume administration and control of digestive symptoms.^5^

Among the preparations used before colonoscopy, polyethylene glycol (PEG), an osmotically balanced laxative, is a widely used bowel preparation.^6^ Concerns has been raised about the use of a split dose preparation of polyethylene glycol‐electrolyte solution (PEG‐ES)‐in which the patient should take part of the solution the evening before colonoscopy and the other part the same day of the procedure, or the conventional whole dose previous day protocol that is accompanied by diet restriction (full fluid diet).^7^ New recommendations stated that the use of split preparation provide a better cleaning level,^8^ for instance a recent study conducted in 2008 showed that split dose group exhibited markedly better degree of bowel cleaning than whole dose group (88.9% vs. 42.6%).^9^ However, some patients report poor bowel cleansing even after the split dose preparation^8^ especially when the procedure is conducted in the afternoon.^10^

Little research has been conducted in Lebanon to compare the split dose and previous day regimen regarding their efficacy and patient compliance. It should be noted that previous study 2 by El Sayed et al.^7^ conducted on Lebanese population relied on subjective terms (excellent to poor score) for the assessment of the preparation's quality, however these terms do not have any official or scientific definitions,^2^ while recent study recommended the use of validated scores to assess the cleansing of the bowel like the Boston Bowel Preparation or Ottawa scales rather than subjective scales.^8^ Based on what has been advanced, we hypothesize that split dosing is more efficacious than whole previous day regimen in cleaning the colon before colonoscopy.

### 1.2 Literature review

#### 1.2.1 Colonoscopy

A common technique for identifying and treating digestive diseases is colonoscopy.^1^, ^11^ An endoscopic examination of the lower gastrointestinal tract.^12^, ^13^, ^14^, ^15^, ^16^, ^17^ The demand for and availability of colonoscopic examinations have expanded in tandem with the steadily rising number of cases of various intestinal illnesses. New techniques for intestinal cleansing, as well as patterns and methods for performing those operations, continue to emerge.^13^, ^14^, ^15^, ^16^, ^17^, ^18^

Diagnostic colonoscopies and, to a greater extent, interventional colonoscopies can hold significant side effects and as a result endager patients lives. An effective and well‐planned endoscopic surgery requires that the bowels be cleaned appropriately.^19^, ^20^, ^21^, ^22^, ^23^, ^24^

However, in an aging culture, there is a far increased likelihood that patients who qualify for endoscopic evaluations and procedures may also have a variety of comorbidities. Polyethylene glycol (PEG) macrogols are most usually used as a way to get ready for surgeries on the large intestine, as well as for diagnostic endoscopic and radiographic testing.^13^, ^14^, ^15^, ^16^, ^17^, ^18^, ^19^, ^25^, ^26^ The intestine must be sufficiently cleansed before an endoscopy, despite the fact that this causes other problems, mostly with the levels of water and electrolytes. Before 2009, patients were typically ready for the exam 1 day before the operation. Two sizable groups of patients getting colonoscopies might be formed. Patients who self‐prepare at home make up the first group.

The other group consists of people who are ill or elderly and have been admitted to the hospital for a variety of conditions. Under the supervision of doctors and nurses, they get ready for colonoscopy procedure in the hospital. The most frequent side effects before, during, and following a colonoscopy are nausea, vomiting, colicky stomach pains, imbalance problems, altered mental status due to head trauma, bone fractures and hemorrhages, and epistaxis.

#### 1.2.2 Colonoscopy procedure

The distal region of the small intestine (terminal ileum), the colon, the rectum, and the anus are all examined during a colonoscopy, which is both a diagnostic and therapeutic treatment. The procedure is carried out with the aid of a colonoscope, a hand‐held flexible tube‐like device with a high‐definition camera attached at the tip transmitting information to the screen and accessory channels used for the cleaning of the mucosa and the lens of colonoscope by inserting fluids and the removal of mucosal lesions.

Over the past few decades, colonoscopy has made CRC a preventable and early detected disease.^27^

#### 1.2.3 Anatomy and physiology of the digestive system

It is very important to keep in mind the anatomy of the digestive system because it affects the performance of colonoscopy and the surgeries especially at the level of the colon.

**Figure 1:**
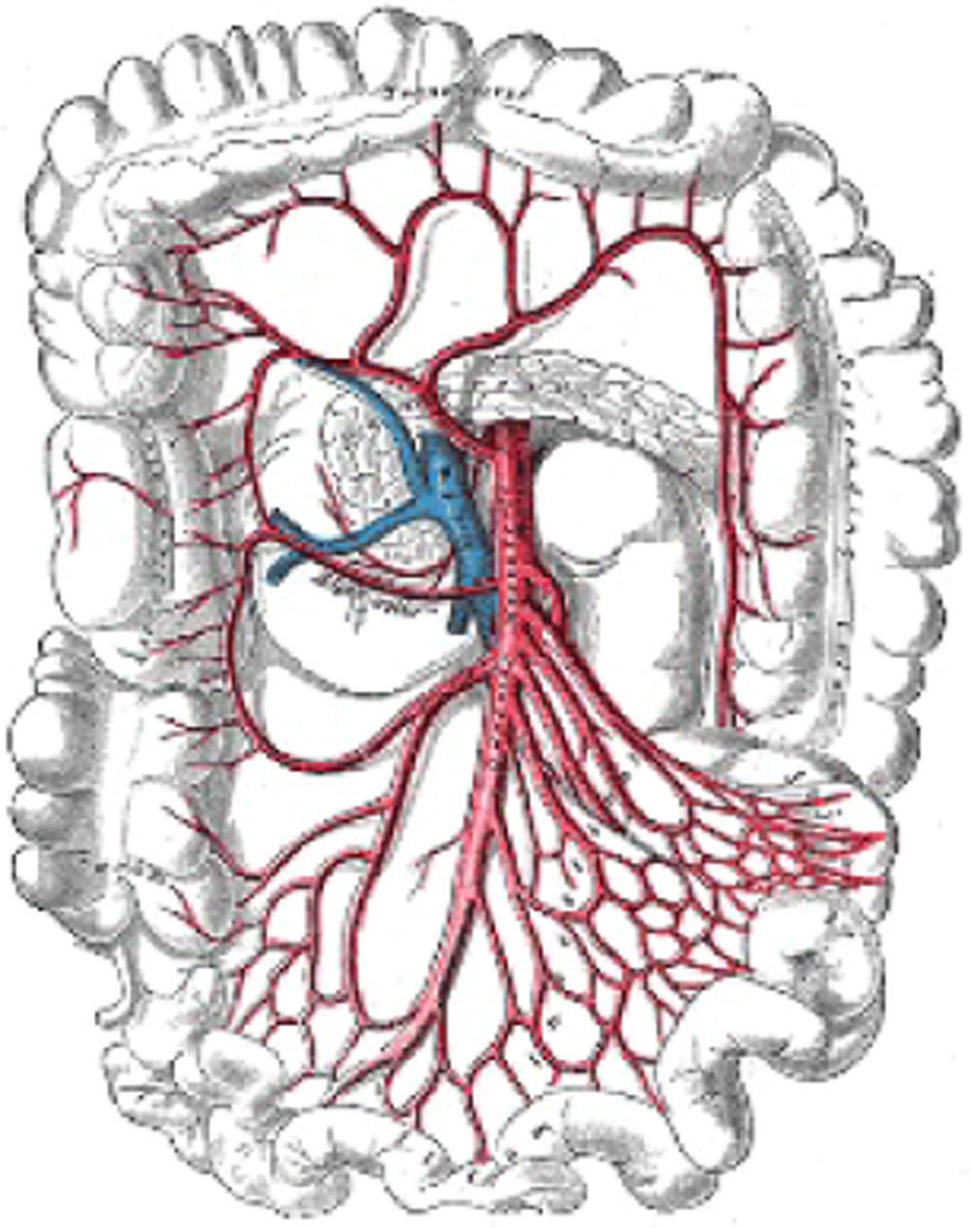
Anatomy of the superior mesentery artery. Descending colon, cacum ilium, and transverse colon. Gray's Anatomy Plates has contributed.^27^

The small and large intestines make up the portion of our digestive system that follows the stomach. The small instestines include respectively the dudenum, jejunum and ileum (Figure 1).

The ileum ends into a pouch like structure known as the cecum, followed by the ascending, transverse, descending colon and finally the sigmoid and rectum giving way to the anal canal.

Although the overall distance varies from male to female, it can be estimated to be between 120 and 160 cm. The ascending colon, hepatic flexure, and transverse colon make up the right colon, whereas the descending colon, splenic flexure, and sigmoid comprise the left colon. The cecum, transverse colon, and descending colon all have diameters of 9, 6, and 3 cm, respectively. The blood supply to the colon is essentially provided by the superior mesenteric artery (SMA) and the inferior mesenteric artery (IMA). The SMA and IMA divide into additional arteries that nourish various parts of the colon (Media 1 and 2). While the IMA supplies from the distal third of the colon to the proximal rectum, the SMA supplies from the small intestines to proximal two thirds of the transverse colon.

Once more, it's crucial to understand when surgical intervention is necessary and appropriate anatomic resections are taken into account.

**Figure 2:**
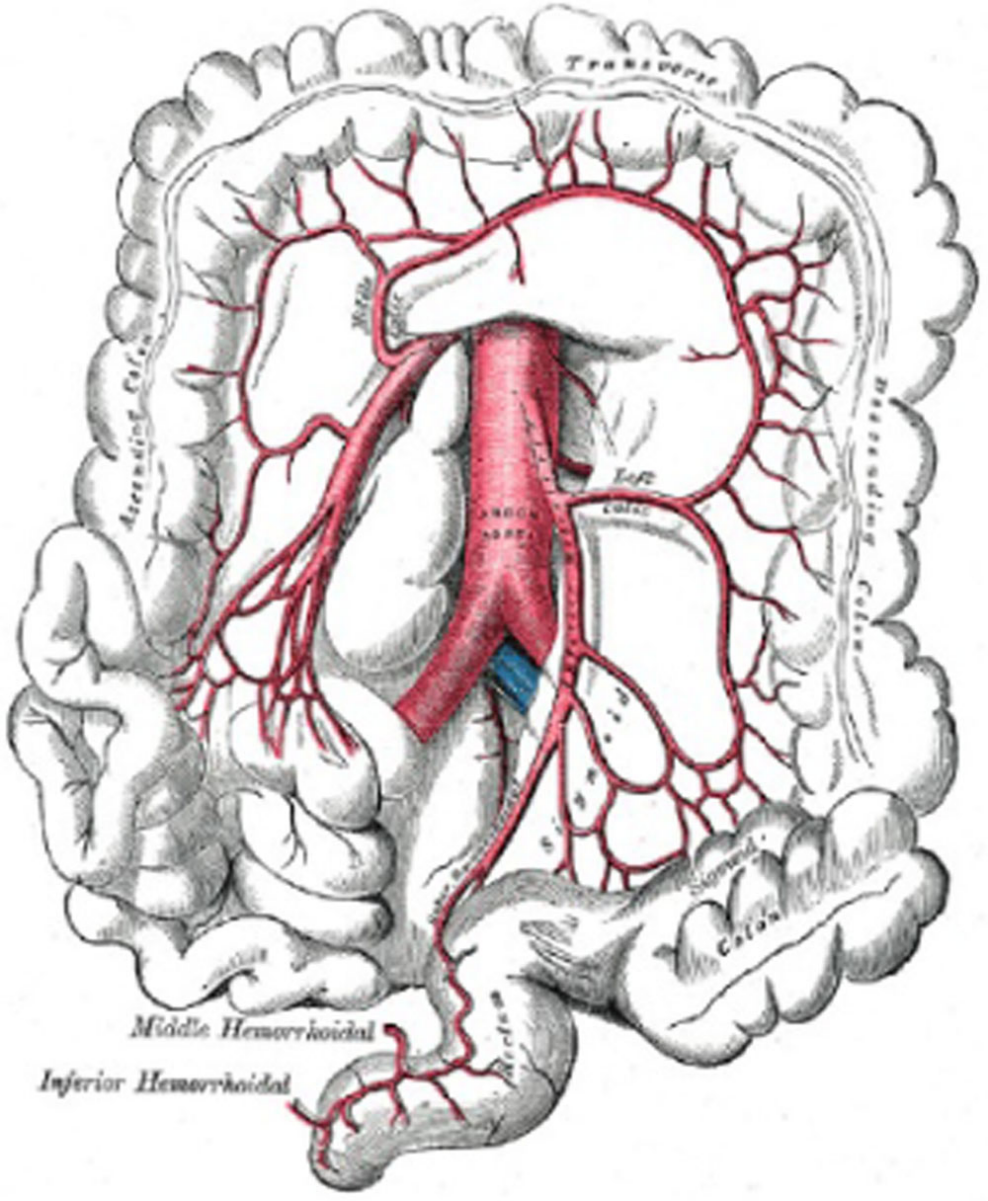
The colon, ascending, transverse, descending, inferior hemorhoidal, and abdominal aorta are branches of the inferior mesenteric artery. Gray's Anatomy Plates has contributed.^27^

Similar to the small intestine, the colon is made up of several layers: Mucosa, Submucosa, Muscularis, and Serosa, which range in thickness from intraluminal to extra luminal (Figure 2).

The way in which the muscularis layers are organized in the large and small intestines differs from one another, among other noticeable differences. Both circular and longitudinal muscle fibers make up the muscularis layer. The longitudinal layer is grouped into three band‐like formations known as the taenia coli, in contrast to the small intestine where these fibers are present present all over the intestine. Additionally, the colon has folds created by many outpouchings known as haustra, these folds can conceal polyps if not adequately observed during colonoscopy.

Regarding colonoscopies, the colon's length and diameter are crucial. The estimation of length, different landmarks and variations aid to direct the location during colonoscopy.^28^

#### 1.2.4 Indication of colonoscopy

Colonoscopies can be done for a variety of reasons. There are two categories of indications: diagnostic and therapeutic. Diagnostic indications can also be divided into screening and elective categories. Depending on the patient's risk, screening colonoscopies are done to check for colorectal malignancies (average vs. high risk). Screening begins at the age of 50 and is carried out every 10 years to check for precancerous or cancerous abnormalities. Surveillance colonoscopies are the subsequent examinations. Earlier surveillance is conducted in accordance with the outcomes of the initial (index) operation.

Before the age of 50, patients with a high risk of developing CRC undergo Colonoscopy, which is then repeated every 1, 2, or 5 years depending on the results. Inflammatory Bowel disease, a family history of CRC before age 60, hereditary polyposis (like Peutz‐Jegher syndrome and familial adenomatous polyposis, resulting from the mutation of an APC gene), LYNCH I, LYNCH II syndromes (nonpolyposis syndromes), and surveillance following CRC resection are a few examples of high risk population.

The American Gastroenterological Association states that screenings for colon cancer should begin at age 40, or 10 years before that age, whichever occurs first, for those with first‐degree relatives who have been diagnosed with the disease.

In addition, known or suspected gastrointestinal bleeding, stool that tests positive for occult blood, inexplicable alterations in bowel habits or patterns, iron deficiency anemia, elderly with weight loss, chronic discomfort of the abdomen, and possible inflammatory bowel illness Other therapeutic indications for colonoscopy are lesions ablation, management of bleeding, strictures dilatation, foreign bodies removal, release of colonic volvulus or megacolon decompression, and therapy of diagnosed neoplasms.^29^, ^30^

The United States Preventive Services Task Force (USPSTF) recommends to do colonoscopy every 10 years for patients with average risk for CRC aged between 50 and 75 years old.^31^ There are other methods for screening, such as fecal immunochemical testing and fecal occult blood tests (FOBT), which are typically reserved for people with an average risk of CRC and are performed annually with flexible sigmoidoscopy every 5 years. These screening methods are not recommended for use in highrisk populations.^32^

#### 1.2.5 Contraindication of colonoscopy

While colonoscopy helps in the diagnosis of acute and chronic pathology, many contraindications exist and have to be taken into account before moving forward with the procedure. Naturally, any procedure cannot be done without the consent of the patient who is willing to undergo and prepare for it. If the patient has little desire to continue, he will not tolerate the bowel preparation. Additionally, patient's medical condition should be taken into account when deciding whether to perform a colonoscopy. This can include diverticulitis, fulminant colitis, ulcerative colitis, toxic megacolon, and more.

In general, if there is inflammation of the bowel, it is better to wait until the eradication of this inflammation.

Colonoscopy is contraindicated in some conditions like:

1. Patient refusal,
2. Recent myocardial infarction,
3. Hemodynamic instability,
4. Peritonitis,
5. Recent surgery with bowel anastomosis,
6. Bowel injury and repair.

Note that the procedure cannot be made before 6 weeks of an acute event.^33^

#### 1.2.6 Complications

There are inherent risks with every invasive procedure that should be made clear to the patient before starting. Generic risks should be discussed during agreement and include general risks ^1^like stroke, reactions to anesthesia, bleeding, heart attack, respiratory difficulty, and death. Patients should be informed that there are hazards associated with colonoscopies, specifically the possibility of bleeding, pain, bloating, rectal rips, pain, infection and intestinal perforation, a more severe but uncommon consequence, estimated to be 0.14%, making colonoscopy a superb low risk tool used to screen for colon cancer.^34^ The Sigmoid colon has the highest risk of perforation. ^1^Shear injury, over distention from excessive insufflation into the colon with weak wall, mechanical polypectomy and electrocautery are the three main ways that can cause perforation.^35^

The colon's turns and corners serve as focal points for the scope to press against as it moves through the colon. It is possible to reduce some of the push and pull forces and reduce the risk of injury by using manipulation techniques that involve the assistant pushing against the abdominal wall. The sigmoid colon has the largest risk of injury because it is the first bend or corner in the large intestine and experiences the most stress as the scope moves toward the cecum.

Post polypectomy electrocoagulation syndrome is another consequence that the practitioner should be aware of, despite its rarity (PPES). Transmural burn is a rare complication of electrocautery that can happen if the practitioner fails to move correctly the tissue. Acute abdominal pain many hours after electrocautery‐assisted polypectomy can occur. Leukocytosis with left shift may be detected in the labs, and a CT scan without symptoms of pneumoperitoneum may reveal a swollen gut and inflammatory alterations. Bowel rest, supportive care, and antibiotics are used to treat it; surgery is not required for resolution. During electrocautery, the doctor can move the polyp away from the colonic wall to lessen the risk of PPES. Additionally, a layer of insulating mucosa can be created by elevating the colonic mucosa away from the intestinal wall by injecting ordinary saline beneath the polyp.^36^

#### 1.2.7 Preparation for colonoscopy

From a patient's perspective, many people experience anxiety and worry about discomfort, preparation, and possible embarrassment when they consider a colonoscopy. But advances in sedative techniques and technology have made the procedure more comfortable. Even though the preparatory steps for colon cleansing may not be pleasurable, strict adherence is essential to its success. Healthcare providers put patients' dignity first and make sure the setting is private and courteous. Mild pain, such as bloating, is normal after a colonoscopy, but major problems are uncommon. Open communication with healthcare providers helps address concerns and ensures a positive experience in this vital preventive healthcare measure.

The majority of patient's biggest complaint about having a colonoscopy is the preparation process, which is also the main barrier to compliance with screening colonoscopies. To avoid the need of repeating colonoscopy targeting the detection of malignant and premalignant lesions an appropriate preparation should be prescribed to the patient. The operation may not be performed if there is insufficient intestinal preparation because of the increased risk of perforation and false‐negative results. Numerous studies demonstrate that using a properly cleansed colon before a colonoscopy greatly improves patient care outcomes and aids in the early detection of cancer. The success of an adequate colonoscopy is marked by this along with a number of other elements, such as the time needed to arrive to the cecum and to withdraw, and the rate of ileocecal intubation.^27^

Simple rules govern proper bowel preparation, such as the requirement that the colon be free of feces and allow for clear vision of the colonic mucosa. Although the idea behind it is straightforward, the actual execution of it can be a challenging and uncomfortable part of a colonoscopy. A variety of drugs and oral laxatives, such as polyethylene glycol, magnesium citrate, magnesium hydroxide, bisacodyl, and sodium picosulfate, can be used in a bowel preparation program. The best bowel cleansing routines have been determined by a number of studies. When selecting the best bowel cleansing plan, though, various aspects must be taken into account. Taste, amount, gastrointestinal discomfort, and efficacy of the item are a few of these. The aim of the Bowklean trial, conducted from October 23, 2013, to March 24, 2014, was to compare the efficacy and patient satisfaction of two different regimens: polyethylene glycol (2–4 L) and Picosulfate/magnesium citrate (300 mL). According to the results of their investigation, ^1^the toleration rate of the picosulfate/magnesium citrate solution is greater than the polyethylene glycol solution and it offered a superior bowel cleanse for optimal colonoscopy visibility.^37^

A clear liquid diet the day before the procedure increases the likelihood of a successful colonoscopy along with the chemical cleansing agent. Patients who are unable to endure a 24 h cleanse might be able to rely on a clear liquid diet for up to 3 days before cleaning the colon with a regimen of little laxative. Enemas can be utilized before a colonoscopy, but by themselves, they do not offer sufficient preparation or a suitable intestinal cleaning. The inability of an enema to reach the transverse colon, ascending colon, and cecum is the main cause of this. They might assist in offering a final cleanse for the rectum and sigmoid colon, but their application is restricted.

There is a debate concerning the use of antibiotics prophylaxis before colonoscopies. While there is no indication for antibiotic prophylaxis, it can be taken into consideration in high risk patients for peritonitis following colonoscopy and polypectomy, such as immunocompromised, receives peritoneal dialysis, and diabetic patients.^38^

Any decision or operation should be fully explained to the patient, and any questions should be addressed, before moving forward. This is the cornerstone of any successful medical practice. It is quite beneficial to sit down and go over the process steps and the advised bowel preparation, particularly with a colonoscopy. Studies have shown that using this teaching strategy rather than just giving out pamphlets or handouts alone results in a more effective bowel preparation. This improves compliance and ensures a successful colonoscopy by directly addressing the patient's expectations and the timetable for bowel preparation.^39^

#### 1.2.8 Guidelines and protocols for colonoscopy

Thorough planning, skillful technique, and good patient communication are essential for a successful colonoscopy. The following useful advice and industry standards can improve the entire experience:

##### Explicit and thorough patient communication

*Detailed information*: Give patients written, understandable information on how to prepare for the procedure as well as what to anticipate throughout it.

Encourage patients to discuss any worries or inquiries they may have in an open discussion before the treatment. An encounter that is more relaxing can be achieved by addressing worries beforehand.

##### Thorough preparation

*Unambiguous instructions*: Stress the importance of closely adhering to the preparation guidelines. A clean, well‐prepared colon is necessary for the procedure to be effective.

*Hydration*: To avoid dehydration and guarantee ideal bowel cleaning, promote a sufficient intake of fluids during the preparatory phase.

Ideally, when preparing for a colonoscopy, it is important to take into account patient variables such as past medical and surgical history, weight and compliance to the preparation.

The patient should be lying down in the left lateral decubitus posture. However, if the situation calls for it, some medical professionals might prefer the patient to be on their back or right side. To protect the bony prominence and to help with positioning, the patient should be lying on their left side with their legs bent and pillows positioned around their head, back, and in the space between their knees.

The endoscopist ought to be in back of the patient. Perform a digital rectal exam (DRE) using a water‐soluble lubricant before inserting the scope. The doctor should now feel the patient for any lumps, bulges, rectocele, or tumors. They should also pay attention to the patient's level of drowsiness, which could have an impact on the anus tone, in addition to the rectal tone. Following the DRE, the clinician should hold the endoscope in their right hand, about 10–20 cm from the working end or lens, with the handle of the scope in their left hand. Again, a substantial amount of water‐soluble lubricant should be applied to assist protect the delicate anus tissue. The endoscope is maneuvered through the various levels of the rectum, colon, and terminal ileum using all of the scope's components (up, down, left, right, and rotation).

The clinician can use certain landmarks to help them locate themselves in the colon. It is vital to keep in mind that the scope can twist and revolve on itself and will not be able to offer a precise measurement farther into the colon, even though typical distances of the anatomy are beneficial.

Important landmarks to keep in mind and take into account when conducting a colonoscopy:

Three separate, larger outpouchings can be found in the initial part of the rectum. This is crucial because it serves as a compartment for fecal storage and enables the human body to go longer intervals between bowel movements.

The sacral protrusion marks the start of the second landmark. This denotes the rectum's proximal end. Because the sacrum bumps up against the colon, it might be trickier to cross; nevertheless, with practice and knowledge, it gets simpler.

As was previously mentioned, taenia coli travels the entire length of the colon; however, these fibers splay out and stop being grouped longitudinally in bands at the beginning of the rectum and the end of the sigmoid colon. Even though these Taenia coli cannot be seen inside the colon, they may usually be detected by the tensile pressures they exert on a bloated gut. They can be used to distinguish between the rectum and the sigmoid colon. The final landmark that can be utilized to pinpoint the location is this one.

Comparatively speaking, the descending colon is rounder than the other parts of the colon. Its diameter, which ranges from 3 to 5 cm, is less than that of the transverse and ascending colon. As the colon continues into the transverse colon, it changes shape from circular to more triangular and its diameter increases. The taenia coli is mostly to blame for this.

Another landmark that can be challenging to get around is the splenic flexure. The endoscope can be pushed past the flexure and into the transverse colon by couterpressing to both quadrants of the right abdomen: the upper and the lower. The assistant should use the Prechel pressure technique, which involves putting pressure with the palm of the hand, as the assistant could get hurt.^40^

It is noticeable that the colon turns blue at the hepatic flexure. It is a reflexion of the liver into the colon, which can indicate to the doctor that he is getting close to the hepatic flexure and moving into the ascending colon. It is significant to highlight that although it is uncommon, excessive pressure in this area can harm the liver.

Compared with the descending and transverse colons, the ascending colon is significantly more dilated. It will keep growing as it approaches the cecum. The ileocecal valve opening, appendiceal entry, taenia coli convergence into a single place called the Mercedes sign, at the end of the cecum, are a few features that aid in locating this site. To help locate the cecum, one should use these markers. Transillumination can aid in supplying more information, but it should not be utilized as a means of arriving at this conclusion.

The endoscopist must feel for any strain or challenges when advancing the colonoscope. If the vision is unclear and the lumen cannot be seen, the scope should never be advanced. Injury risk is increased as a result.

The endoscopist should try to intubate the ileocecal valve to aid finish the colonoscopy, prevent any missed lesions, and provide more evidence that the cecum has been reached. This aids in confirming that the cecum was truly accessed, as well as aiding in the visualization of the ileum and the diagnosis of inflammatory bowel disease.

As the endoscopist enters into the colon, he will evaluate for any mucosal lesion like a polyp and remove it. The lesion is colored with methylene blue or India ink, and it should be removed surgically if found to be large.

The clinician ought to be inspecting every part of the colon during withdrawal and peering behind every haustral fold to find polyps even the tiniest ones, and if polyps are not properly detected, they may cause a colonoscopy to come out falsely negative and cause cancer to be ignored. As was previously discussed, various indicators are utilized to determine whether a colonoscopy was effective. For a colonoscopy to be deemed successful and to give the patient the proper screening, the clinician should withdraw from the colon for at least 6–8 min. There is a higher chance of missing polyps and developing early cancer when the scope is removed more quickly than this.^41^

To improve patient recovery, reduce flatulence, and ease discomfort from distention, it is essential to aspirate part of the insufflation when withdrawing from the colon. The anus and the dentate line should be checked for lesions or hemorrhoids near the end of the colonoscopy by retroflecting the scope.

### 1.3 Objectives

#### 1.3.1 Primary objective

We aim to assess the efficacy of a split dose of PEG versus the traditional previous day regimen in cleaning the colon before colonoscopy using Boston bowel preparation scale, to direct gastroenterologists toward the best preparation.

#### 1.3.2 Secondary objective

We evaluated patient satisfaction regarding the modality of bowel preparation applied based on the main side effects experienced by the patients (nausea, vomiting, cramps, sleep disturbances…), in addition polyp detection rate (PDR) was estimated.

## 2 PART II: SUBJECTS AND METHODS

### 2.1 Ethical considerations

The study proposal was submitted to the Institutional Review Board (IRB) at Al Sahel General Hospital and Al Zahraa Hospital University Medical Center. The IRB was approved and access was granted to recruit patients (Supporting Information: Appendix 1 and 2).

Top confidentiality had been applied and the patients' names were replaced by study codes. Each group had a specific survey (Supporting Information: Appendix 3–6). An informed consent will be obtained from all patients enrolled in our study before data collection. It includes a thorough explanation of the study's objectives and procedure as well as the purpose of the questionnaire. Patients will be told that they have the right to withdraw from the research at any time if they wish to do so (Supporting Information: Appendix 6).

### 2.2 Study design and population

We conducted a 3 years prospective, multicentric, parallel, trial during the years 2021–2023. Hospitalized patients undergoing elective colonoscopy at Sahel General Hospital and Al Zahraa Hospital University Medical Center (ZHUMC) were enrolled in this study after being eligible to participate according to the inclusion and exclusion criteria mentioned above.

Patients will be randomly assigned to receive one of the two regimens before the colonoscopy:

- Group A: will be given the preparation as a split dose: 1st dose on the day before the procedure and 2nd dose on the same day of the procedure.
- Group B: will be given the preparation as a whole dose on the previous day, along with full fluid diet.

#### 2.2.1 Inclusion criteria

The subjects included in the study were:

1. Age >18 years.
2. Undergoing a routine elective colonoscopy.

#### 2.2.2 Exclusion criteria

The subjects excluded were:

1. Age <18 years.
2. Chronic constipation and laxative dependent.
3. Significant gastroparesis.
4. Gastric outlet obstruction.
5. Allergy to PEG.
6. Refusal to participate.

#### 2.2.3 Sample size

The study of Abdul‐Baki et al. demonstrated that the split‐dose preparation provided better cleansing than the whole‐dose preparation (88.9% vs. 42.6%, *p* < 0.001).^9^

Using the G‐Power calculator, *Z* test concluded that the minimum sample size should be 20 in the study group and 20 in the group control (using alpha error 5% and power 95%). It is highly recommended to use a big N to have accurate results.

### 2.3 Data collection

Patients were randomly allocated to two arms. Split dose regimen group will be instructed to take two sachets of PEG preparation dissolved in 2 L of water on the day before the procedure (between 6:00 p.m. and 10:00 p.m.), followed by two sachets dissolved in 2 L of water on the same day of the procedure (between 6:00 a.m. and 8:00 a.m.). Colonoscopy for this group will be scheduled at 11:00 a.m. Previous day regimen group will be instructed to take four sachets of PEG preparation dissolved in 4 L of water on the day before the procedure (between 4:00 p.m. and 11:00 p.m.) and to strictly adhere to a diet containing only soup, Jello, Custard, Yogurt, Labneh without bread and filtered juice on the preceding day. Colonoscopy for this group will be scheduled between 8:00 a.m. and 10:00 a.m. Patients will then be asked to fill a paper‐based questionnaire that includes information about patient's age, gender, colonoscopy indication, side effects experienced after completing the 4 sachets required (cramps, stomach pain, headache, nausea, vomiting, sleep disturbances, bloating and general malaise), patient satisfaction using a scale of 1 to 4 (1 = not satisfied at all, 2 = not satisfied, 3 = a little bit satisfied, 4 = satisfied) and the willingness to take the same preparation in the next colonoscopy with justification. (Both written instructions and questionnaire will be given to the patient) (Supporting Information: Appendix 3–6). Instructions will be provided by the nurse or fellow not by the gastroenterologists who will be blinded to which group the patients belonged to; furthermore, each patient will receive a specific number.

Bowel cleansing will be evaluated by the endoscopists using Boston Bowel Preparation Scale (BBPS); each segment (right colon, transverse and left colon) will be rated as: 0 = mucosa not seen, 1 = only portion of mucosa is seen, 2 = mucosa is well seen, 3 = entire mucosa is well seen with no residual staining, stool fragments or opaque liquid. Total score range from minimum 0 (very poor) to maximum 9 (excellent).^17^ In addition, PDR will be evaluated as: (number of examinations with polyps/total number of examinations) × 100, moreover polyps found will be described and mentioned as single or multiple (Supporting Information: Appendix 3–6). Each patient will give his number to the gastroenterologist concerned to fill this premade sheet, once colonoscopy is completed.

### 2.4 Data analysis

All statistical analysis was performed using IBM SPSS version 25.

Data will be presented and compared between the two groups. Demographic data (age and gender), indication, side effects, patient satisfaction scale, willingness to take the same preparation in the next colonoscopy), and BBPS will be compared between the two groups. The following tests will be used in the analysis:

1. *Mann–Whitney test*: It is used to test the difference between the two groups in function of a continuous variable which is not normally distributed (e.g., BBPS, patient satisfaction scale).
2. *χ^2^ and Fisher test*: used to test the difference between the two groups in function of a categorical variable (e.g., gender, scale, willingness to take the same preparation in the next colonoscopy).

A difference is considered statistically significant if the *p* value is less than 0.05 (Alpha error 5%).

## 3 PART III: RESULTS

Patients were distributed into two groups:

1. 98/200 (49%) in Group A (patients treated with PEG preparation as a split dose: 1st dose on the day before the procedure and 2nd dose on the same day of the procedure).
2. 102/200 (51%) in Group B (patients treated with PEG preparation as a whole dose on the previous day, along with full fluid diet).

### 3.1 Indication of colonoscopy

Indications of colonoscopy are presented in function of the two study groups and set in Table 1. The following indications were shown in the study population, from a total of 200 patients, noting surveillance (2%), screening (8.5%), bleeding (34%), change in bowel habits (constipation/diarrhea) (32%), inflammation (10.5%), pain (12%), and anemia (1.5%). There was no statistically significant difference between the two study groups and the indications (*p* > 0.05).

**Table 1:** Indication of colonoscopy in function of the two study groups.

		Groups		Total	*p* Value
		Group A (*N* = 98)	Group B (*N* = 102)		
Surveillance	No	98	98	196	0.12^b^
		100.0%	96.1%	98.0%	
	Yes	0	4	4	
		0.0%	3.9%	2.0%	
Screening	No	89	94	183	0.73^a^
		90.8%	92.2%	91.5%	
	Yes	9	8	17	
		9.2%	7.8%	8.5%	
Bleeding	No	66	66	132	0.69^a^
		67.3%	64.7%	66.0%	
	Yes	32	36	68	
		32.7%	35.3%	34.0%	
Change in bowel habits (constipation/diarrhea)	No	64	72	136	0.42^a^
		65.3%	70.6%	68.0%	
	Yes	34	30	64	
		34.7%	29.4%	32.0%	
Inflammation	No	89	90	179	0.55^a^
		90.8%	88.2%	89.5%	
	Yes	9	12	21	
		9.2%	11.8%	10.5%	
Pain	No	84	92	176	0.33^a^
		85.7%	90.2%	88.0%	
	Yes	14	10	24	
		14.3%	9.8%	12.0%	
Anemia	No	97	100	197	>0.99^b^
		99.0%	98.0%	98.5%	
	Yes	1	2	3	
		1.0%	2.0%	1.5%	

*Note*: Tests done using *χ* ^2^ test (a) and Fisher exact test (b).

Among the 98 patients in Group A, 9 (9.2%) underwent colonoscopy for screening, 32 (32.7%) underwent colonoscopy for bleeding, 34 (34.7%) did colonoscopy for change in bowel habits (constipation/diarrhea), 9 (9.2%) underwent colonoscopy for inflammation, 14 (14.3%) did colonoscopy for pain and 1 (1%) had colonoscopy for anemia.

Among the 102 patients in Group B, 4 (3.9%) underwent colonoscopy for surveillance, 8 (7.8%) underwent colonoscopy for screening, 36 (35.3%) underwent colonoscopy for bleeding, 30 (29.4%) did colonoscopy for change in bowel habits (constipation/diarrhea), 12 (11.8%) underwent colonoscopy for inflammation, 10 (9.8%) did colonoscopy for pain and 2 (2%) had colonoscopy for anemia.

### 3.2 Demographic characteristics

Demographic characteristics are presented in function of the two study groups and set in Table 2. Patients were distributed between 105/200 (52.5%) males and 95/200 (47.5%) females. Patients were aged between 18 and 30 years (17.5%), 31 and 50 years (48.5%), and more than 50 years (34%). There was no statistically significant difference between the two study groups and the demographic characteristics (*p* > 0.05).

**Table 2:** Demographic characteristics in function of the two study groups.

	Groups		Total	*p* Value
	Group A (*N* = 98)	Group B (*N* = 102)		
Age				0.53
18–30 years	20	15	35	
	20.4%	14.7%	17.5%	
31–40 years	19	21	40	
	19.4%	20.6%	20.0%	
41–50 years	23	34	57	
	23.5%	33.3%	28.5%	
51–60 years	25	23	48	
	25.5%	22.5%	24.0%	
61 years or older	11	9	20	
	11.2%	8.8%	10.0%	
Gender				0.68
Male	50	55	105	
	51.0%	53.9%	52.5%	
Female	48	47	95	
	49.0%	46.1%	47.5%	

*Note*: Tests done using *χ* ^2^ test.

Patients in group A were distributed between 50/98 (51%) males and 48/98 (49%) females. Patients were aged between 18 and 30 years (20.4%), 31 and 50 years (42.9%), and more than 50 years (36.7%).

Patients in group B were distributed between 55/102 (53.9%) males and 47/102 (46.1%) females. Patients were aged between 18 and 30 years (14.7%), 31 and 50 years (53.9%), and more than 50 years (31.3%).

### 3.3 Adverse events

**Figure 3:**
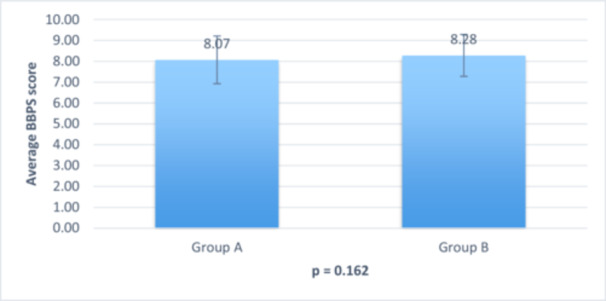
BBPS in function of the two study groups.

Adverse events are presented in function of the two study groups and set in Table 3 and (Figure 3). The following adverse events were shown in the study population noting cramps 97/200 (48.5%), stomach ache 64/200 (32%), headache 62/200 (31%), vomiting 106/200 (53%), nausea 106/200 (53%), sleep disturbance 54/200 (27%), bloating 53/200 (26.5%) and malaise 52/200 (26%). Results showed that Sleep disturbance was shown in 20.4% of patients in group A and in 33.3% of patients in group B, the only statistically significant different side effect between the two groups (*p* = 0.04).

**Table 3:** Adverse events in function of the two study groups.

		Groups		Total	*p* Value
		Group A (*N* = 98)	Group B (*N* = 102)		
Cramps	No	46	57	103	0.21
		46.9%	55.9%	51.5%	
	Yes	52	45	97	
		53.1%	44.1%	48.5%	
Stomach ache	No	62	74	136	0.16
		63.3%	72.5%	68.0%	
	Yes	36	28	64	
		36.7%	27.5%	32.0%	
Headache	No	68	70	138	0.91
		69.4%	68.6%	69.0%	
	Yes	30	32	62	
		30.6%	31.4%	31.0%	
Vomiting	No	52	42	94	0.09
		53.1%	41.2%	47.0%	
	Yes	46	60	106	
		46.9%	58.8%	53.0%	
Nausea	No	47	47	94	0.79
		48.0%	46.1%	47.0%	
	Yes	51	55	106	
		52.0%	53.9%	53.0%	
Sleep disturbance	No	78	68	146	**0.04**
		79.6%	66.7%	73.0%	
	Yes	20	34	54	
		20.4%	33.3%	27.0%	
Bloating	No	74	73	147	0.53
		75.5%	71.6%	73.5%	
	Yes	24	29	53	
		24.5%	28.4%	26.5%	
Malaise	No	69	79	148	0.26
		70.4%	77.5%	74.0%	
	Yes	29	23	52	
		29.6%	22.5%	26.0%	

*Note*: Tests done using *χ* ^2^ test. The *p*‐value of 0.04 indicates that there is a 4% probability that the observed difference in sleep disturbance between the two groups occurred by chance alone, assuming that there is no real difference between the groups.

### 3.4 Patients' satisfaction

Patients' satisfaction characteristics are presented in function of the two study groups and set in Table 4. Out of the 200 patients, 38% were satisfied with the preparation followed for the colonoscopy, 41.5% were a little bit satisfied with the preparation followed for the colonoscopy, and 20.5% were not satisfied with the preparation followed for the colonoscopy. There was no statistically significant difference between the two study groups and patients' satisfaction with the preparation followed for the colonoscopy (*p* = 0.9) where the average satisfaction score was 3.02 ± 1.03 over 4 in Group A patients and 3.04 ± 0.99 over 4 in Group B patients.

**Table 4:** Patients' satisfaction in function of the two study groups.

		Groups		Total	*p* Value
		Group A (*N* = 98)	Group B (*N* = 102)		
How satisfied are you with the preparation you followed for the colonoscopy?	Not satisfied at all	15	14	29	0.97^a^
		15.3%	13.7%	14.5%	
	Not satisfied	6	6	12	
		6.1%	5.9%	6.0%	
	A little bit satisfied	39	44	83	
		39.8%	43.1%	41.5%	
	Satisfied	38	38	76	
		38.8%	37.3%	38.0%	
Satisfaction	Median (IQR)	53 (28)	26 (31)	52.5 (59)	0.9^b^
	Min–Max	1–4	1–4	1–4	
Are you going to follow the same preparation for the next colonoscopy?	No	29	22	51	0.19^a^
		29.6%	21.6%	25.5%	
	Yes	69	80	149	
		70.4%	78.4%	74.5%	
Reasons for not going to follow	Impossible to tolerate the preparation	0	1	1	–
		0.0%	1.0%	0.5%	
the same preparation for the next colonoscopy	Intolerance of the amount of water and the medication	1	0	1	
		1.0%	0.0%	0.5%	
	Severe vomiting	0	1	1	
		0.0%	1.0%	0.5%	
	Severe vomiting and diarrhea	1	0	1	
		1.0%	0.0%	0.5%	
	The medication is disgusting	1	0	1	
		1.0%	0.0%	0.5%	
	The medication is very annoying	3	1	4	
		3.1%	1.0%	2.0%	
	The preparation is very annoying	5	2	7	
		5.1%	2.0%	3.5%	

*Note*: Tests done using *χ* ^2^ test (a) and independent *t* test (b).

Abbreviation: IQR, interquartile range.

Among the 98 patients in Group A, 70.4% will go to follow the same preparation for the next colonoscopy. The top two reasons for not going to follow the same preparation for the next colonoscopy in group A patients were the preparation is very annoying (5.1%) and the medication is very annoying (3.1%).

Among the 102 patients in Group B, 78.4% will go to follow the same preparation for the next colonoscopy. The top reason for not going to follow the same preparation for the next colonoscopy in group B patients was the preparation is very annoying (2%).

### 3.5 Boston Bowel Preparation Scale

Boston bowel preparation scale was used to assess the efficacy of the preparation in the two study groups. Before washing or suctioning, each segment is scored on a scale of 0–4. Results of BBPS items were shown in Table 5. There was no statistically significant difference between the two study groups in function BBPS items and score (*p* > 0.05).

**Table 5:** BBPS in function of the two study groups.

	Groups		Total	*p* Value
	Group A (*N* = 98)	Group B (*N* = 102)		
Left colon (descending and sigmoid colon, and rectum)				0.95^a^
1	1	1	2	
	1.0%	1.0%	1.0%	
2	26	25	51	
	26.5%	24.5%	25.5%	
3	71	76	147	
	72.4%	74.5%	73.5%	
Transverse (includes hepatic and splenic flexures)				0.2^a^
1	3	0	3	
	3.1%	0.0%	1.5%	
2	21	21	42	
	21.4%	20.6%	21.0%	
3	74	81	155	
	75.5%	79.4%	77.5%	
Right colon (cecum and ascending colon)				0.07^a^
1	2	3	5	
	2.0%	2.9%	2.5%	
2	32	19	51	
	32.7%	18.6%	25.5%	
3	64	80	144	
	65.3%	78.4%	72.0%	
Boston Bowel Preparation Scale (BBPS)				0.16^b^
Median (IQR)	26 (65)	21 (76)	51 (141.5)	
Min ‐ Max	5–9	5–9	5–9	

*Note*: Tests done using *χ* ^2^ test (a) and independent *t* test (b).

Abbreviation: IQR, interquartile range.

There was no statistically significant difference between the two study groups and BBPS (*p* = 0.16) where the average BBPS was 8.07 ± 1.14 in Group A patients and 8.28 ± 1.0 in Group B patients (Figure 3).

### 3.6 Polyp detection

Polyp detection was shown in 83/200 (41.5%) of the study population, 41.8% in Group A patients, and 41.2% in Group B patients, with no statistically significant difference (*p* = 0.925) (Table 6).

**Table 6:** Polyp detection in function of the two study groups.

	Groups		Total	*p* Value
	Group A (N = 98)	Group B (N = 102)		
Polyp detection				0.93
No	57	60	117	
	58.2%	58.8%	58.5%	
Yes	41	42	83	
	41.8%	41.2%	41.5%	
Type of polyp				0.38
Single	29	34	63	
	70.7%	79.1%	75.0%	
Multiple	12	9	21	
	29.3%	20.9%	25.0%	

*Note*: Tests done using *χ* ^2^ test.

In the study population, the types of polyps were single polyp 63/83 (75%) and multiple 21/83 (25%) with no statistically significant difference between the study groups and the type of polyps (*p* = 0.38).

## 4 PART IV: DISCUSSION

### 4.1 Discussion

Traditionally, the complete bowel‐cleansing preparation is administered in the evening before the colonoscopy. And, to avoid sleep disruption, it must be administered early in the evening, resulting in poor bowel cleansing reported as a 70% sufficient cleaning rate,^42^ which was much lower in our center at 50% because we only utilized low‐volume 2 L PEG. The main cause of poor bowel cleanliness could be the long‐time interval between the last preparation intake and the time of the colonoscopy, which was supposed to be limited to 4–6 h. To meet the recommended interval time for the morning colonoscopy, patients had to get up at dawn.

Poor compliance and unhappiness would result. Previous research has indicated that split preparation provides better quality and compliance when compared with SID preparation, however due to little data, it is debatable if it is still true for morning colonoscopies and low volume 2 L PEG.

We hypothesized that the SPD regimen would increase compliance and colon cleansing since it delays the intake of PEG in the early morning. We compared split dose: 1st dose on the day before the procedure and 2nd dose during samemorning to a whole dose on the previous day, along with full fluid diet.

Colonoscopy indications (monitoring, screening, bleeding, change in bowel habits (constipation/diarrhea, inflammation, discomfort, and anemia) are described in relation to the two study groups. No statistically significant difference was detected concerning the indications of the two trial groups; this was relevant with another study conducted in 2020 where it shows no difference in terms of indication.^43^

In this study, there was no variation in bowel preparation based on age or gender. Some research have shown that being elderly is beneficial,^44^, ^45^ but in males they were related to inadequate preparation, including in people from the Middle East and Asia.^8^ However, studies are carried out on either an inpatient or outpatient basis, and it is widely recognized that inpatient bowel preparation is associated with poor bowel preparation when compared with outpatient scenarios.^45^ And this could be due to many factors as the condition and the comorbidities of the hospitalized or the elderly patient that can contribute to the effect of poor bowel preparation, and if not taken into considerations could lead to these results.

Moreover, adverse events including cramps, stomachache, headache, Nausea, Vomiting, Bloating and Malaise shows no statistical significance between Group A and B. Similarly, there were no recorded adverse effects that resulted in withdrawal, thus there were no statistically significant differences between groups. According to another study, the whole‐dose group included more patients who experienced nausea and vomiting. Other negative effects were comparable between the two groups. The bowel preparation in the whole‐dose group showed a higher rate of discomfort.^5^ Although sleep disruption was considerably different in our findings between the two groups, Group A lower than Group B, other findings showed that patients afflicted by sleep disturbance were equivalent in the two groups.^43^ The morning preparation has the advantage of interfering less with the patient's routines and work schedules, due to the fact that the dose of the day before the procedure can be given early in the evening which will not cause a sleep disturbance; Patients frequently complain about difficulty sleeping after taking the evening preparation.^5^

There was no statistically significant difference in function of BBPS items and scores between the two study groups, nor between the study groups and the detection of polyps. Another study revealed no correlation between transverse colon segment scores and polyp detection. The transverse colon had fewer absolute polyps than the right and left colons, possibly reducing the potential to detect substantial changes. Because there is no correlation between transverse colon segment scores and polyp detection, a simplified BBPS that simply involves assessments of the right and left colon is possible.^46^

Significant advancements and new technology in the field of gastroenterology have been brought to light in relation to colonoscopies, especially with regard to bowel preparations. The application of artificial intelligence (AI) algorithms to improve the effectiveness and caliber of bowel cleansing before colonoscopy examinations is one notable development. The effectiveness of an AI‐driven approach in streamlining bowel preparation processes was shown in a study published in the Journal of Gastrointestinal Endoscopy.^47^ This led to enhanced colon visualization and higher rates of colorectal lesion diagnosis.

Furthermore, efforts have been made to improve patient comfort and compliance by developing innovative bowel preparation formulations. A novel oral solution demonstrated encouraging results in producing better bowel cleansing with lower volume requirements in a clinical trial that examined the product.^48^ These latest developments highlight the continuous attempts to improve colonoscopy methods to guarantee more precise and patient‐friendly operations.

### 4.2 Impact of the study

Traditionally, gastroenterologists relied on previous day whole preparation to achieve a good cleaning of the bowel before the colonoscopy, however taking such high doses in a limited time interval can be disturbing for patients. If the split preparation turned out to be more efficient in cleaning the colon with less side effects reported by the patients, it could be used from now on as a modality of choice by the physicians so that a better colonic visualization is achieved with the highest degree of patient compliance. Once patients are more satisfied and comfortable, they will be more adherent to their routine procedure, therefore detection of precancerous lesions and polyps and their removal could lead to decreased CRC incidence and mortality.

### 4.3 Study limitations

The fact that this was a single‐center study was one of the study's shortcomings. When asked about tolerability and preferred treatment, patients in general favored the split‐dose treatment, although no assessment instrument was utilized to determine this. Because the trial was done as an outpatient and the endoscopy department had a busy schedule, we did not employ a specific questionnaire to assess patient tolerance and compliance with both regimens. More research in this area is needed to compare different regimens in our community and to discover predictors of adequate bowel preparation to minimize incomplete colonoscopies.

### 4.4 Study perspectives

The study's strength comes from the fact that both groups had a reasonably large number of patients, and their beginning features were similar. Lebanon's populations have several comorbidities (e.g., diabetes and HTN), allowing us to better explore the predictors of bowel preparation. Both groups had the same endoscopists.

## 5 PART V: CONCLUSION

In conclusion, if the procedure is scheduled in the morning, split evening‐morning dose showed better colon cleansing than morning‐only dosing. Effective, safe, and dependable bowel preparation solutions are becoming more widely available, albeit the most pleasant options continue to be the most expensive.

With the split dose administration and a greater understanding of the appropriate interval between the preparation and colonoscopy, efficacy has improved.

The major Gastrointestinal Societies have agreed that the agent of choice should be selected depending on the condition of each patient, but that a split dosage regimen can be suggested in all circumstances.

More research is needed to help doctors choose the best regimen for their patients, depending on their age and comorbid diseases. However, low volume PEG preparations appear to be the most ideal preparatory agents, given their good tolerance, effectiveness, safety in patients with comorbid diseases and low price.

Many interventions to improve patient tolerability and adherence are evaluated in ongoing studies, like patient education about preprocedural preparation and smart‐phone based applications to schedule the doses of the preparation. These interventions will increase both patient and endoscopist satisfaction.

## AUTHOR CONTRIBUTIONS

**Blaybel Sara**: Conceptualization; data curation; investigation; methodology; writing—review and editing. **Hammoud Ghinwa**: Conceptualization; data curation; investigation; methodology; writing—review and editing. **Mourda Layla**: Conceptualization; data curation; investigation; methodology; writing—review and editing. **Hallal Mahmoud**: Supervision. **Khalil Ali**: Supervision. **Mckey Remy**: Conceptualization; data curation; investigation; methodology; writing—review and editing. All authors have read and approved the final version of the manuscript, corresponding author had full access to all of the data in this study and takes complete responsibility for the integrity of the data and the accuracy of the data analysis.

## CONFLICT OF INTEREST STATEMENT

The authors declare no conflict of interest.

## TRANSPARENCY STATEMENT

The lead author Mckey Remy affirms that this manuscript is an honest, accurate, and transparent account of the study being reported; that no important aspects of the study have been omitted; and that any discrepancies from the study as planned (and, if relevant, registered) have been explained.

## Supporting information

Supporting information.

Supporting information.

## Data Availability

The data that supports the findings of this study are available in the supplementary material of this article.
