# Supplementary material for: Split doses versus whole dose bowel preparation using polyethylene glycol for colonoscopy: A multicentric prospective Lebanese randomized trial between 2021 and 2023
Source: Health Sci Rep. 2024 Apr 22;7(4):e2047. doi: 10.1002/hsr2.2047 (PMC11035750; doi:10.1002/hsr2.2047)
Supplement: Supplementary file 1 — Supporting information. [file HSR2-7-e2047-s002.docx]

# Supplementary material

# Database.xlsx.

# APPENDICES

**Appendix 1: IRB approval from Al Sahel General Hospital**


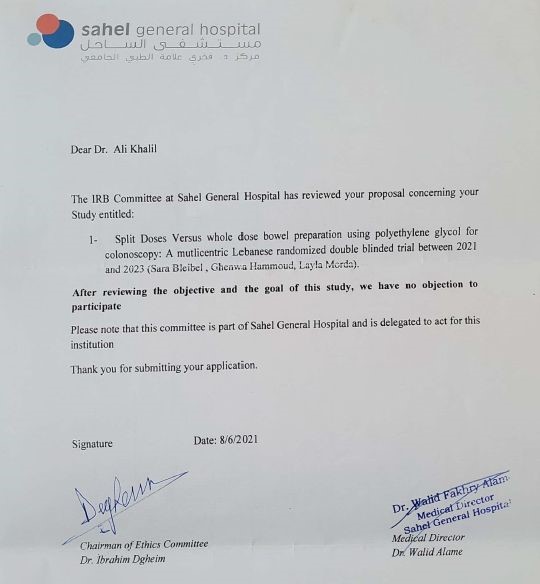


**Appendix 2: IRB approval from Al Zahraa Hospital University Medical Center**


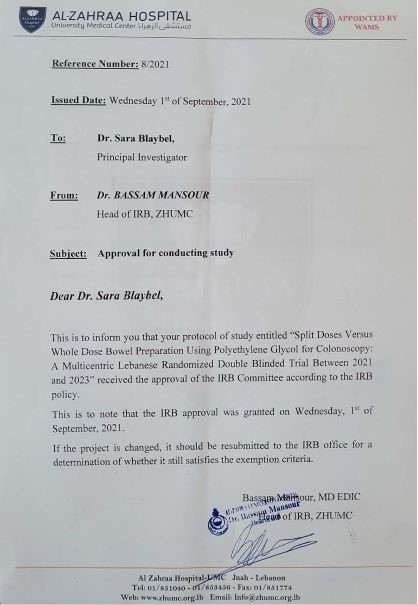


**Appendix 3: Data Collection Form for Group A (English Form)**

Group A

Preparation 1:

Before colonoscopy, the patient should adhere to the following instructions:

- Take 3 sachets of PEG preparation dissolved in 3 liters of water, from 6:00 PM to 10:00 PM on theday before the colonoscopy.
- Take one sachet of PEG preparation dissolved in 1 liter of water, from 6:00 AM to 8:00 AM on the same day of the colonoscopy.

*The colonoscopy will be scheduled around 11:00 AM.*

**If you are 18 years old or older and interested in participating in a study to evaluate the efficacy of pre-colonoscopic preparation followed by the patient and its impact on the result of colonoscopy, please fill out this form before the procedure and give it to the nurse or resident.**

**It will not take more than 2 minutes.**

**We are concerned to inform you that all information will remain confidential and will be used only for scientific purposes.**

**Your refusal to participate in the study does not in any way deprive you of your legal rights or exempt the doctor from his/her professional or legal responsibilities.**

**Select the appropriate answer:**

- Which preparation have you followed?

Preparation 1

Preparation 2

- Indication of colonoscopy:

Surveillance

Screening

Bleeding

Change in bowel habits (constipation/diarrhea)

Inflammation

Pain

Other

- Age:

18-30

31- 40

41-50

51-60

61 or older

- Gender:

Male

Female

- Have you noticed any of the side effects mentioned below after taking your preparation?

Yes No

- 1. Cramps
  2. Stomach ache
  3. Headache
  4. Vomiting
  5. Nausea
  6. Sleep disturbance
  7. Bloating
  8. Malaise
- How satisfied are you with the preparation you followed for the colonoscopy?

Not satisfied at all

Not satisfied

A little bit satisfied Satisfied

- Are you going to follow the same preparation for the next colonoscopy?

Yes Why?: ……………………………………………………………………………….. No Why?: ………………………………………………………………………………..

**Appendix 4: Data Collection Form for Group B (English Form)**

Group B

Preparation 2:

Before colonoscopy, the patient should adhere to the following instructions:

- During the 24 hours before colonoscopy, he should follow a soft diet including exclusive sorts of food: Maggi soup, Jello, Custard, Laban, Labneh (without bread), filtered juice.
- Take 4 sachets of PEG preparation dissolved in 4 liters of water, from 4:00 PM to11:00 PM on the day before the colonoscopy.

*The colonoscopy will be scheduled between 8:00AM and 10:00 AM.*

**If you are 18 years old or older and interested in participating in a study to evaluate the efficacy of pre-colonoscopic preparation followed by the patient and its impact on the result of colonoscopy, please fill out this form before the procedure and give it to the nurse or resident.**

**It will not take more than 2 minutes.**

**We are concerned to inform you that all information will remain confidential and will be used only for scientific purposes.**

**Your refusal to participate in the study does not in any way deprive you of your legal rights or exempt the doctor from his/her professional or legal responsibilities.**

**Select the appropriate answer:**

- Which preparation have you followed?

Preparation 1

Preparation 2

- Indication of colonoscopy:

Surveillance

Screening

Bleeding

Change in bowel habits (constipation/diarrhea)

Inflammation

Pain

Other

- Age:

18-30

31- 40

41-50

51-60

61 or older

- Gender:

Male

Female

- Have you noticed any of the side effects mentioned below after taking your preparation?

Yes No

1.Cramps

2.Stomach ache

3.Headache

4.Vomiting

5.Nausea

6.Sleep disturbance

7.Bloating

8.Malaise

- How satisfied are you with the preparation you followed for the colonoscopy?

Not satisfied at all

Not satisfied

A little bit satisfied Satisfied

- Are you going to follow the same preparation for the next colonoscopy?

Yes Why?: ……………………………………………………………………………….. No Why?: ………………………………………………………………………………..

**Appendix 5: Data Collection during endoscopy**

Name of Endoscopist:

Patient’s No:

● **Boston Bowel Preparation scale (BBPS)**

Score 0: Unprepared colon segment with mucosa not seen due to solid stool that cannot be cleared.

Score 1: portion of the mucosa of the colon segment is seen, but other areas of the colon segment are not seen well due to staining, residual stool, and/or opaque liquid.

Score 2: minor amount of residual staining, small fragments of stool, and/or opaque liquid are visible, but the mucosa of the colon segment are seen well.

Score 3: The entire mucosa of the colon segment is seen well with no residual staining, small fragments of stool, or opaque liquid.


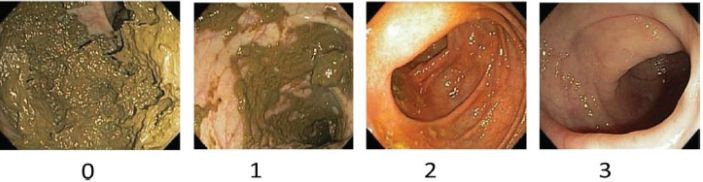


**Bowel cleanliness score:**

Left colon (descending and sigmoid colon, and rectum): 0 1 2 3

Transverse (includes hepatic and splenic flexures): 0 1 2 3 Right colon (cecum and ascending colon): 0 1 2 3

**Total score:** ……………...

● **Polyp detection:**

Yes**:** Single

Multiple

N**o**

**Appendix 6: Consent form**

INFORMED CONSENT FORM FOR PARTICIPATION IN A SCIENTIFIC STUDY PATIENT INFORMATION:

Dear patient This document aims to request your consent to participate in one of the medical studies conducted by a group of students at the Faculty of Medical Sciences - Lebanese University. It also includes a review of the details of the study. Please read the document thoroughly and consult your doctor if there are any unclear details.

PURPOSE OF THE STUDY: This study aims to evaluate the effectiveness of the pre colonoscopic preparation followed by the patient, and its impact on the quality of the colonoscopy and the patient’s comfort, in order to guide doctors toward the best preparation that allows for accurate diagnosis of colon diseases while also being the most suitable for the patient in terms of quality of life and side effects.

PROCEDURE: The patient follows the instructions mentioned in a form marked by a specific number that the nurse or resident gives him on the day before the colonoscopy. Before going to the clinic the next day, the patient fills out the whole form and returns it to the person who gave it to him. After performing the colonoscopy, the doctor fills out a chart with the same form number to assess the cleanliness of the colon and the ability to see the mucosa.

CONFIDENTIALITY: The patient will be identified by a number without knowing his identity, thus the colonoscopy result will be matched with the form marked by the same number. Only those who are conducting the study will have access to the data, which is mainly identified by numbers rather than names, with utmost care for the patient's privacy.

VOLUNTARY PARTICIPATION: Participation in this medical study is completely voluntary, and you can withdraw from it at any time. Refusing to participate would have no negative consequences. If you have any concerns about the questions on the form or the medication prescribed, do not hesitate to contact the nurse or resident who gave you the form.

PATIENT CONSENT: This is a declaration that I am willing to take part in the aforementioned study, I acknowledge that the nature of the study and its objectives were explained to me in a straightforward and comprehensive manner by the doctor treating me. I agree that I have carefully read and understood the above text, that the doctor has answered all of my questions, and that I have been informed of my right to withdraw at any time.

Patient’s Signature
